# Machine Learning-Driven Insights in Cancer Metabolomics: From Subtyping to Biomarker Discovery and Prognostic Modeling

**DOI:** 10.3390/metabo15080514

**Published:** 2025-08-01

**Authors:** Amr Elguoshy, Hend Zedan, Suguru Saito

**Affiliations:** 1 Biofluid Biomarker Center, Graduate School of Medical and Dental Sciences, Niigata University, Niigata 9502181, Japan; 2 Graduate School of Science and Technology, Niigata University, Niigata 9502181, Japan; 3 Department of Biomedical Sciences, Cedars-Sinai Medical Center, Los Angeles, CA 90048, USA

**Keywords:** cancer metabolomics, machine learning (ML), biomarker discovery, tumor subtyping, prognostic modeling

## Abstract

Cancer metabolic reprogramming plays a critical role in tumor progression and therapeutic resistance, underscoring the need for advanced analytical strategies. Metabolomics, leveraging mass spectrometry and nuclear magnetic resonance (NMR) spectroscopy, offers a comprehensive and functional readout of tumor biochemistry. By enabling both targeted metabolite quantification and untargeted profiling, metabolomics captures the dynamic metabolic alterations associated with cancer. The integration of metabolomics with machine learning (ML) approaches further enhances the interpretation of these complex, high-dimensional datasets, providing powerful insights into cancer biology from biomarker discovery to therapeutic targeting. This review systematically examines the transformative role of ML in cancer metabolomics. We discuss how various ML methodologies—including supervised algorithms (e.g., Support Vector Machine, Random Forest), unsupervised techniques (e.g., Principal Component Analysis, t-SNE), and deep learning frameworks—are advancing cancer research. Specifically, we highlight three major applications of ML–metabolomics integration: (1) cancer subtyping, exemplified by the use of Similarity Network Fusion (SNF) and LASSO regression to classify triple-negative breast cancer into subtypes with distinct survival outcomes; (2) biomarker discovery, where Random Forest and Partial Least Squares Discriminant Analysis (PLS-DA) models have achieved >90% accuracy in detecting breast and colorectal cancers through biofluid metabolomics; and (3) prognostic modeling, demonstrated by the identification of race-specific metabolic signatures in breast cancer and the prediction of clinical outcomes in lung and ovarian cancers. Beyond these areas, we explore applications across prostate, thyroid, and pancreatic cancers, where ML-driven metabolomics is contributing to earlier detection, improved risk stratification, and personalized treatment planning. We also address critical challenges, including issues of data quality (e.g., batch effects, missing values), model interpretability, and barriers to clinical translation. Emerging solutions, such as explainable artificial intelligence (XAI) approaches and standardized multi-omics integration pipelines, are discussed as pathways to overcome these hurdles. By synthesizing recent advances, this review illustrates how ML-enhanced metabolomics bridges the gap between fundamental cancer metabolism research and clinical application, offering new avenues for precision oncology through improved diagnosis, prognosis, and tailored therapeutic strategies.

## 1. Introduction

Cancer has a multifaceted aspect representatively characterized by metabolic reprogramming, which plays a critical role in tumor progression, drug resistance, and patient prognosis [1]. Metabolomics, the comprehensive study of metabolites in biological systems, has emerged as a powerful tool for uncovering the metabolic alterations associated with cancer [2]. By capturing the dynamic metabolic landscape in the tumors, metabolomics provides valuable insights into cancer biology, enabling the identification of diagnostic biomarkers, therapeutic targets, and prognostic signatures [3]. However, the inherent complexity and high dimensionality of metabolomics data often lead to substantial analytical challenges and variability between datasets. Machine learning (ML), with its capacity to manage large-scale data, uncover hidden patterns, and generate accurate predictions, has emerged as an essential tool for improving the robustness and reliability of metabolomics analyses. Notably, ML techniques have shown remarkable progress in cancer metabolomics, enabling precise cancer subtyping, biomarker discovery, and the development of prognostic models with high specificity [4].

This review focuses on the integration of machine learning (ML) with metabolomics to address key challenges in cancer research. We start exploring how ML constitutes the algorithms, such as Similarity Network Fusion (SNF), the adaptive Least Absolute Shrinkage and Selection Operator (LASSO), and Support Vector Machines (SVMs), that have been applied to metabolomic data to refine cancer subtyping. For example, studies on triple-negative breast cancer (TNBC) have utilized metabolomic profiling and ML to classify tumors into distinct metabolic subtypes, revealing associations with relapse-free survival and treatment responses [5]. These findings highlight the potential of ML to uncover metabolic heterogeneity and guide personalized treatment strategies. Next, we discuss the role of ML in biomarker discovery, where metabolomics data have been leveraged to identify metabolic signatures that distinguish cancer from benign conditions and predict treatment efficacy. For instance, studies have employed ML models, such as Random Forest and Partial Least Squares Discriminant Analysis (PLS-DA), to identify key metabolites associated with breast cancer and colorectal cancer, achieving high diagnostic accuracy and predictive performance [6,7]. These advancements underscore the potential of ML-driven metabolomics to enhance the possibility of early detection and monitor the efficacy of the treatment.

In the realm of prognostic modeling, ML has been instrumental in uncovering metabolic disparities across racial groups and predicting cancer outcomes. For example, metabolomics studies have identified distinct metabolic signatures in African American and Non-Hispanic White breast cancer patients, providing insights into the metabolic and epigenetic factors influencing prognosis [8]. Similarly, ML models have been used to predict survival outcomes in lung and ovarian cancers based on metabolomics data, demonstrating the potential of ML to improve risk stratification and patient management [9,10].

Beyond breast cancer, this review highlights the broader applications of ML in metabolomics for other types of cancers. In colorectal cancer, ML has enhanced early diagnosis and classification through the analysis of salivary and serum metabolomic data [11,12]. In ovarian cancer, ML-driven metabolomics has enabled the identification of diagnostic biomarkers in plasma extracellular vesicles, offering new avenues for non-invasive screening [10]. Similarly, in prostate, thyroid, and pancreatic cancers, machine learning (ML) has been employed to develop diagnostic models and classify disease subtypes, underscoring its versatility and broad applicability across a range of cancer types [13,14,15]. By accumulating the latest findings, this review aims to provide a comprehensive overview of how machine learning is transforming cancer metabolomics research, from subtyping and biomarker discovery to prognostic modeling. We also discuss the challenges and opportunities in this rapidly evolving field, emphasizing the need for robust validation, integration of multi-omics data, and translation of findings into clinical practice. Through this exploration, we hope to underscore the transformative potential of ML-driven metabolomics in advancing cancer research and improving patient outcomes.

## 2. Overview of Metabolomics

Metabolomics is the comprehensive study of small molecules, known as metabolites, within cells, biofluids, tissues, or organisms. These metabolites, which include amino acids, lipids, sugars, and nucleotides, represent the intermediates or end products of cellular processes that are closer to the phenotype and provide a snapshot of the physiological state of a cell or organism at a given time [16,17,18]. Unlike transcriptomics and proteomics, which focus on mRNA and protein expression (intermediate steps in the central dogma), metabolomics captures the downstream products of gene expression, providing a more direct understanding of cellular function and biochemical characteristics. This makes metabolomics a powerful tool for elucidating the complete behavior of the analyzed samples [19].

Metabolomic analyses employ high-throughput technologies such as nuclear magnetic resonance (NMR) spectroscopy and mass spectrometry (MS). The choice of platform significantly influences study outcomes, as each method has distinct advantages and limitations. NMR offers high-throughput, nondestructive analysis with minimal sample preparation, quantitative accuracy without internal standards, and redundant spectral information for robust metabolite identification. However, NMR is limited to detecting relatively abundant metabolites (≥1 μM), whereas MS provides far higher sensitivity (femtomolar to attomolar range), resolution (~10^3^–10^4^), and dynamic range (~10^3^–10^4^) [20].

Despite its sensitivity, MS detects only metabolites that ionize efficiently, leaving ~40% of known metabolites unobservable. Ion suppression—where matrix effects (e.g., co-eluting compounds, contaminants) inhibit ionization of target metabolites—further reduces detection reliability. To mitigate these issues, MS-based metabolomics typically couples chromatography (GC-MS, LC-MS) to separate metabolites before analysis. However, chromatography introduces its own biases, including metabolite degradation during derivatization, variable column recovery, and retention time misalignment across replicates [21].

In contrast, NMR avoids ionization- or chromatography-related artifacts but struggles with low-abundance metabolites. Thus, combining NMR and MS can offset their individual weaknesses: NMR validates MS identifications and quantifies dominant metabolites, while MS expands coverage of low-concentration species. Together, these platforms enable a more comprehensive view of the metabolic landscape in cancer.

Metabolomics studies are generally categorized into targeted and untargeted (global) approaches, each serving distinct purposes in metabolite analysis.

Targeted metabolomics focuses on the identification and quantification of a predefined set of known metabolites, typically ranging from tens to hundreds. This approach is commonly applied in clinical studies requiring the precise measurement of specific biomarkers. By focusing on listed metabolites, targeted metabolomics offers high sensitivity, accuracy, and reproducibility, making it ideal for validating hypotheses or monitoring metabolic changes related to specific pathways or diseases [22].

Data acquisition methods in targeted metabolomics include the following: (1). Multiple Reaction Monitoring (MRM/SRM), typically performed on triple–quadrupole instruments. This method selects specific precursor-fragment ion transitions for each metabolite, providing excellent quantitative precision, sensitivity, and reproducibility in large-scale targeted assays [23]. (2). Parallel Reaction Monitoring (PRM) is implemented on high-resolution MS platforms like quadrupole-Orbitraps and monitors full MS^2^ spectra of each selected precursor. PRM offers enhanced selectivity and post-acquisition flexibility, enabling high-confidence quantification—these methods have quantified over 200 metabolites with superior performance [24]. (3). Pseudotargeted approaches combine Q TOF untargeted data acquisition with triple–quadrupole MRM quantification. These hybrid strategies have enabled monitoring of hundreds of metabolites (e.g., 854 MRM pairs) with both untargeted coverage and targeted reliability [25].

In contrast, untargeted metabolomics aims to profile as many metabolites as possible, including both known and unknown compounds. This hypothesis-generating approach allows for comprehensive exploration of metabolic changes across different biological states. The data generated from untargeted studies can be used for relative quantification across the sample groups and to identify novel metabolic alterations that may warrant further investigation using targeted methods.

There are two primary data acquisition strategies in untargeted metabolomics: data-dependent acquisition (DDA) and data-independent acquisition (DIA) [26]. In DDA, a full scan MS1 is first performed to obtain accurate mass measurements for all detectable molecules (referred to as features). Based on signal intensity, a subset of the most abundant metabolites is then selected for MS/MS fragmentation to generate detailed spectral data [27,28]. This method, commonly used in proteomics, allows for high-quality fragmentation spectra but may overlook low-abundance metabolites due to its focus on the most intense signals. Alternatively, DIA captures MS1 and MS/MS data for all precursor ions, regardless of their abundance. This approach can be executed through methods like MSE, which fragments all ions simultaneously, or sequential windowed acquisition of all theoretical fragment ions (SWATH), which fragments ions in predefined mass ranges. While DIA provides more comprehensive coverage compared to DDA, it produces complex spectra where fragment ions are not directly linked to their precursor ions [29,30]. Advanced data analysis techniques are required to match fragments with their corresponding precursors based on mass-to-charge ratio (*m*/*z*), retention time (RT), and, when applicable, drift time (DT). Both DDA and DIA ultimately generate rich datasets that describe metabolites using these key features. In the final identification step, precursor and fragment ions are searched against metabolite databases to assign accurate identities, enabling researchers to interpret metabolic changes within biological systems.

## 3. Overview of Machine Learning Analysis in Cancer Metabolomics

ML has emerged as a transformative tool in cancer metabolomics, enabling researchers to extract meaningful insights from complicated high-dimensional datasets. By leveraging computational algorithms, ML can identify patterns, classify samples, and predict outcomes based on metabolic profiles, offering new avenues for understanding cancer biology and improving clinical outcomes. The integration of ML with metabolomics is particularly powerful because it can increase data accuracy by eliminating several background factors [31,32]. This section provides an overview of the role of ML in cancer metabolomics, highlighting its applications and methodologies, specifically focusing on preprocessing techniques and model evaluation and validation approaches.

Metabolomics generates a huge amount of data, often accompanied by high dimensionality, noise, and complex interactions between metabolites, all of which have the potential to cause a misleading result. Traditional statistical methods may not be able to solve the issues in such datasets, particularly when dealing with nonlinear relationships or interactions between metabolites. In contrast, ML algorithms are particularly well-suited for addressing these complexities. ML can process high-dimensional data, handle multiple variables simultaneously, and uncover hidden patterns, enabling robust classification of samples (e.g., tumor vs. normal tissue, cancer subtypes), identification of biomarkers associated with cancer progression, treatment response, and patient outcomes, as well as the prediction of clinical endpoints such as survival and recurrence. Furthermore, ML facilitates the reconstruction of metabolic networks, providing insights into disease biology. This ML-driven phenotyping represents a powerful approach for elucidating cancer-associated metabolic alterations and translating metabolomics findings into clinically actionable insights. Recent practical applications underscore the growing indispensability of ML in cancer metabolomics research [4,6,14,33,34,35].

### 3.1. Preprocessing Analysis Approaches and Techniques

Preprocessing is a critical step in metabolomics data analysis, as raw data often contain noise, missing values, and batch effects, all of which can compromise the performance of machine learning models. Effective preprocessing aims at minimizing instrumental artifacts and irrelevant biological variability to ensure that the data are clean, normalized, and suitable for downstream analysis. The first step in preprocessing typically involves data cleaning and imputation [36,37]. Metabolomics data frequently contain missing values due to technical limitations or low metabolite abundance [38]. Common imputation methods include mean or median imputation, k-nearest neighbors (k-NNs), and multivariate approaches such as Multiple Imputation by Chained Equations (MICEs) [39,40]. Noise reduction techniques, such as smoothing filters or wavelet transforms, are also applied to improve data quality [41,42].

Following data cleaning, normalization and scaling are essential to correct for variations in sample concentration or instrument sensitivity. Normalization methods ensure fair comparison between samples by correcting for variations in sample quantity and composition. This can occur pre-acquisition, where sample amounts are adjusted before analysis using markers like creatinine (for urine) or cell counts, though this relies on accurate reference markers—which some samples lack. Post-acquisition normalization adjusts mass spectrometry signals after data collection using methods like sum, median, probabilistic quotient normalization (PQN), maximal density fold change (MDFC), and quantile normalization [43].

Scaling methods like autoscaling, Pareto scaling, range scaling, and vast scaling adjust metabolomics data to reduce differences in absolute abundance, ensuring that all metabolites contribute equally to the analysis and enabling better comparison [44,45].

Another critical preprocessing step is batch effect correction, which addresses variations introduced by differences in sample preparation or instrument performance [46]. Methods like ComBat are commonly used to remove batch effects while preserving biological variability [47,48].

Finally, feature selection and dimensionality reduction are employed to reduce the number of metabolites to the most relevant ones, improving model performance and interpretability. Feature selection techniques, such as univariate analysis, recursive feature elimination (RFE) [49], or the adaptive Least Absolute Shrinkage and Selection Operator (LASSO) regression [50], identify key metabolites associated with the biological question of interest [51]. Dimensionality reduction methods, such as Principal Component Analysis (PCA) [52], t-Distributed Stochastic Neighbor Embedding (t-SNE) [53], or Uniform Manifold Approximation and Projection (UMAP) [54], transform high-dimensional data into a lower-dimensional space while preserving its structure. These preprocessing steps collectively ensure that the data are robust and suitable for downstream ML analysis.

### 3.2. Common Machine Learning Approaches in Cancer Metabolomics

ML approaches in cancer metabolomics can be broadly categorized into supervised learning, unsupervised learning, and deep learning, each serving distinct purposes [55]. Supervised learning is used for classification and regression tasks, with algorithms such as Support Vector Machines (SVMs), Random Forests, Logistic Regression, and Neural Networks. These models are particularly effective for differentiating cancer types, predicting patient outcomes based on metabolic profiles, and identifying biomarkers associated with specific phenotypes [56]. In contrast, unsupervised learning is used for clustering and dimensionality reduction, with algorithms such as Principal Component Analysis (PCA) [52], t-Distributed Stochastic Neighbor Embedding (t-SNE) [53], and K-Means Clustering [57]. These methods are invaluable for identifying metabolic subtypes of cancer and discovering novel biomarkers. Deep learning, which includes algorithms such as Convolutional Neural Networks (CNNs) [58] and Recurrent Neural Networks (RNNs) [59], is used for complex pattern recognition in large datasets, such as analyzing spectral data from mass spectrometry (MS) or nuclear magnetic resonance (NMR) and integrating multi-omics data. Each of them has unique strengths and can be chosen based on the specific research purpose and dataset characteristics.

### 3.3. Model Evaluation and Validation Approaches

After preprocessing and model training, robust evaluation and validation are essential to ensure the reliability and generalizability of ML models. The first step in model evaluation involves selecting appropriate evaluation metrics based on the task at hand. For classification tasks, metrics such as accuracy, precision, recall, F1 score, and the area under the receiver operating characteristic curve (AUC-ROC) are commonly used. For regression tasks, metrics such as the mean squared error (MSE) and the coefficient of determination (R^2^) provide insights into model performance. These metrics help assess the model’s ability to make accurate predictions and distinguish between different classes or outcomes [60].

To ensure that the model’s performance is not overfitted to the training data, cross-validation techniques are employed. K-fold cross-validation, where the dataset is split into k subsets and the model is trained and validated k times, is a widely used approach. Leave-one-out cross-validation (LOOCV), a special case of k-fold cross-validation, is particularly useful for small datasets. Stratified cross-validation ensures that each fold maintains the same proportion of classes as the original dataset, providing a more reliable estimate of model performance. However, cross-validation alone is not sufficient to assess generalizability. External validation on an independent dataset is crucial to confirm the model’s robustness. Public datasets, such as those from The Cancer Genome Atlas (TCGA) or Gene Expression Omnibus (GEO), or multi-center studies can be used for this purpose [61].

**Figure 1:**
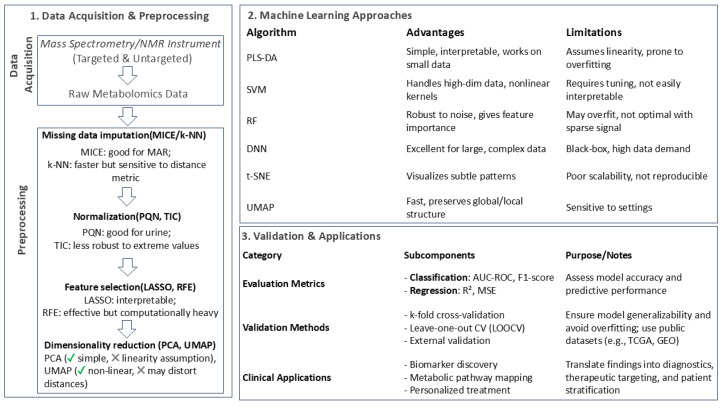
A Computational framework for cancer metabolomics: from data acquisition to clinical applications via machine learning. √ indicates pros, and × indicates cons.

In addition to validation, robustness testing is performed to assess the stability of the model. Permutation testing, where class labels are shuffled to evaluate the significance of model performance, and bootstrapping, where the dataset is resampled with replacement to estimate variability, are commonly used techniques. These tests help ensure that the model’s performance is not due to chance or overfitting. Finally, interpretability and explainability are critical for translating machine learning findings into biological insights. Feature importance methods, such as SHAP (SHapley Additive exPlanations) or LIME (Local Interpretable Model-Agnostic Explanations), identify the most influential metabolites in the model’s predictions [62]. Pathway analysis further maps significant metabolites to biological pathways, providing a functional context for the results (Figure 1; Table S2).

### 3.4. Strengths and Limitations of ML Methods in Cancer Metabolomics

While machine learning has shown great promise in cancer metabolomics, it is important to critically evaluate the strengths and limitations of various algorithms to ensure optimal application.

Partial Least Squares Discriminant Analysis (PLS-DA) is widely used for metabolomics classification due to its ability to handle multicollinearity, noisy data, and small sample sizes. Its interpretability and relatively simple implementation make it a popular choice. However, PLS-DA assumes linear relationships and may underperform when nonlinear dependencies dominate the dataset [63,64].

Support Vector Machines (SVMs) are effective in handling high-dimensional data and modeling nonlinear relationships or combinatorial relations in the data through kernel functions. SVM is robust and much more resistant to overfitting. It often outperforms simpler linear models, especially when sufficient labeled data are available. However, it requires extensive hyperparameter tuning and may lack interpretability in biomedical contexts [65].

Random Forest (RF) provides an ensemble-based approach that can capture nonlinear interactions and is relatively robust to noise, missing values, and variable scaling, and it provides internal validity measures and feature importance, aiding biomarker discovery. In several cases, RF outperformed PLS, SVM, and LDA in clinical metabolomics [66]. Nonetheless, RF may underperform in datasets with sparse signal-to-noise ratios or small numbers of informative features; the risk of overfitting depends on tree count and depth.

Artificial Neural Networks (ANNs) and Deep Learning Methods: Although their applicability to metabolomics studies has rarely been explored, deep neural networks (DNNs) offer strong capabilities for modeling complex nonlinear patterns and achieving high predictive accuracy; however, their use is limited by high data requirements and low interpretability—limitations that have been addressed by modified approaches such as ensemble DNNs (EDNNs) for improved regression performance [67] and DNN-MDA models that incorporate variable importance estimation for enhanced biomarker discovery [68].

Unsupervised learning techniques are widely used in metabolomics to uncover intrinsic data structures without prior labeling. Principal Component Analysis (PCA) is the most commonly applied method, valued for its simplicity and ability to reduce dimensionality while preserving variance. PCA is particularly effective in preliminary data exploration, helping to identify outliers, detect clustering patterns, and assess batch effects in untargeted metabolomics studies. However, PCA assumes linear relationships and may not capture complex, nonlinear structures that are often present in biological systems [54,69] (Table S2).

To address these limitations, more advanced nonlinear dimensionality reduction methods such as t-distributed stochastic neighbor embedding (t-SNE) and Uniform Manifold Approximation and Projection (UMAP) have been increasingly adopted. t-SNE is effective at preserving local data structure and visualizing subtle class separations in metabolomics data, though it is sensitive to parameter settings and lacks reproducibility. In contrast, UMAP offers faster computation, better preservation of global and local data structures, and improved scalability, making it well-suited for large-scale metabolomics datasets. Both methods have shown value in clustering metabolic phenotypes and visualizing sample groupings based on underlying metabolic profiles [54,70].

A comparative analysis across eight algorithms on ten clinical metabolomics datasets found that data quality and sample size had greater impact than algorithm type; nonlinear methods (e.g., RF, SVM) offered minimal performance improvements over PLS-DA [71] (Table S2).

In evaluating the utility of machine learning approaches in cancer metabolomics, we emphasize that method selection should be guided by the characteristics of the dataset (e.g., dimensionality, sample size, noise level) and the specific research objective (e.g., classification, biomarker discovery, prediction). For instance, PLS-DA is a valuable starting point in small-sample studies due to its interpretability and ease of implementation, though it requires careful cross-validation to avoid overfitting. Random Forest is highly versatile and performs robustly across varied metabolomics datasets, especially when dealing with missing values and complex interactions. Support Vector Machines excel in high-dimensional classification tasks but may require computationally intensive tuning. In contrast, deep learning models, such as DNNs and CNNs, offer superior predictive power for large datasets and integrative analyses but remain limited by their “black-box” nature and high data demands. Unsupervised techniques like PCA, t-SNE, and UMAP are indispensable for exploratory data visualization and subgroup discovery, though they do not directly support outcome prediction. As such, we encourage researchers to adopt a hybrid approach, where unsupervised techniques assist in data structure exploration, while supervised models are applied for robust classification or regression tasks. Our analysis supports the view that no single ML model is universally optimal, and success in metabolomics applications depends on a well-informed, context-specific modeling strategy (Figure 1).

## 4. Machine Learning-Driven Insights in Breast Cancer: From Subtyping to Biomarker Discovery and Prognostic Modeling

### 4.1. Subtyping and Classification of Breast Cancer

Machine learning (ML) has played a transformative role in breast cancer (BC) subtyping, particularly for triple-negative breast cancer (TNBC), a clinically heterogeneous subtype lacking targeted therapies. One comprehensive study applied metabolomic profiling to 330 triple-negative breast cancer (TNBC) tumor samples and 149 paired normal tissues using LC-MS-based polar metabolomics and lipidomics, identifying significant metabolic alterations. Using supervised and unsupervised machine learning methods, the researchers classified TNBC into three metabolic subtypes (C1, C2, and C3). Statistical analysis (Mann–Whitney U test and KEGG pathway analysis) was able to identify 452 dysregulated metabolites and highlighted dysregulated metabolic pathways considering the KEGG database. A Similarity Network Fusion (SNF) algorithm was employed for unsupervised clustering, revealing metabolic heterogeneity among TNBC subtypes. For supervised classification, the least absolute shrinkage and selection operator (LASSO) and support vector machine (SVM) models were trained using bootstrapped discovery and test cohorts to classify basal-like immune-suppressed (BLIS) tumors into C2 and C3 metabolic subtypes, demonstrating strong predictive performance via Receiver Operating Characteristic (ROC) curve analysis with AUC = 0.89 using six metabolites. Key outcomes included metabolic heterogeneity, prognostic stratification, and therapeutic targets (sphingosine-1-phosphate (S1P) for C1; N-acetyl-aspartyl-glutamate (NAAG) for C2). Notably, the C2 subtype was linked to worse relapse-free survival (RFS) [5] (Tables S1 and S3). Despite these advances, limitations remain: the SVM model’s lack of interpretability and unvalidated prognostic relevance of subtype C3 underscore the need for hybrid models incorporating explainable AI (e.g., SHAP) and external validation.

In a complementary study, CPSI-MS and DESI-MSI were employed to profile metabolites in TNBC tissues and serum. A total of 150 samples (75 triple-negative breast cancer (TNBC); 75 precancerous normal tissue (PNT)) were used for training, and 90 samples (45 TNBC; 45 PNT) were used for testing. Statistical filtering identified 76 metabolites. The downregulated species included asparagine (Asn), sphingosine 1-phosphate (S1P), S-adenosylhomocysteine (SAH), cytidine, thymidine, glutamine (Gln), and fatty acids, whereas the up-regulated species included glutamate (Glu), proline (Pro), leucine (Leu), citrulline, hypoxanthine (Hypo), serine phosphate (Ser-P), and acyl carnitines. ML (Lasso regression) achieved high accuracy (AUC 0.96) using 22 tissue and 15 serum metabolites, validated via DESI-MSI and independent cohorts. Partial Least Squares Discriminant Analysis (PLS-DA) was applied for unsupervised clustering, showing clear separation between TNBC and PNT. Key metabolites like glutamate and acylcarnitines highlighted altered energy metabolism, while pathway analysis revealed glutaminolysis and lipid remodeling as hallmarks of TNBC [72]. Integration of spatial metabolomics via DESI-MSI enhanced diagnostic resolution and serum panels offered promise for non-invasive screening. Nevertheless, the small cohort size and reliance on linear models (e.g., PLS-DA) warrant validation using nonlinear classifiers like Random Forest (RF) in broader cohorts.

### 4.2. Biomarker Discovery and Treatment Response Prediction

Several studies have demonstrated the utility of ML in identifying diagnostic and pharmacodynamic biomarkers through metabolomics. In one investigation, a multimodal omics approach coupled with machine learning was employed to identify plasma biomarkers for early-stage breast cancer detection. Plasma samples from 13 cancer and 18 benign patients (discovery set) and 5 cancer and 7 benign patients (validation set) were analyzed using LC-MS/MS-based metabolomics and lipidomics. Data preprocessing included missing value imputation, K-nearest neighbor (k-NN), locally weighted scatterplot smoothing (LOWESS) normalization, and Pareto scaling. Univariate analysis identified 73 biomarker candidates, with 7 forming a composite signature (e.g., glutamate, ether-linked phosphatidylcholine (PC(O-)), sphingomyelin (SM)). Machine learning workflows included unsupervised PCA (revealing minimal group separation) and supervised PLS-DA (limited predictive ability: Q2 < 0.4). However, multivariate models (linear SVM, random forest) using the refined signature achieved exceptional performance (AUC > 0.98) in validation. Key metabolites like glutamate and glycochenodeoxycholate were upregulated in cancer, aligning with known tumor metabolism dysregulation (e.g., glutaminolysis). Ether-linked lipids (PC(O-)) and sphingomyelins (SMs) showed diagnostic potential [6] (Tables S1 and S3). However, inconsistencies between training and validation performance and a lack of deep learning models or feature attribution tools limited the interpretability and generalizability of findings.

In another study, metabolomics and machine learning were integrated to identify pharmacodynamic biomarkers for inetetamab in HER2+ breast cancer. Plasma from 23 patients underwent LC-MS analysis (9 control and 14 inetetamab-treated) using machine learning to identify metabolic biomarkers. A total of 6889 metabolites were detected, with 289 key differential metabolites identified through univariate and multivariate statistical analyses, including PCA, hierarchical clustering, and Orthogonal Partial Least Squares Discriminant Analysis (OPLS-DA). Feature selection was conducted using Logistic Regression, Support Vector Machine, and Random Forest, with SHAP values used to select the top 20 biomarkers. Logistic Regression showed AUCs of 0.995 (training) and 0.780 (validation), while SVM and RF exhibited AUCs of 0.995 across both sets. The final biomarkers, FAPy-adenine and 2-Pyrocatechuic acid, showed a significant reduction after inetetamab treatment and were negatively correlated with treatment duration [73].

These results demonstrate ML’s capability to detect treatment-induced metabolic shifts. The use of SHAP promotes interpretability alongside high predictive accuracy. However, discrepancies between training and validation highlight overfitting risks and the need for external validation.

A third study deployed an optimized ML pipeline for BC prediction using plasma metabolomics data from 185 cancer cases and 99 matched controls. Data preprocessing included propensity score matching to correct class imbalance and three feature selection methods (SVC, PCA, and Naive Bayes (NB)) to refine predictive biomarkers. An optimized Support Vector Classifier with a radial basis function (RBF) kernel served as the primary supervised algorithm, achieving an AUC of 99% and accuracy of 95%. PCA was employed for dimensionality reduction, identifying 50 key features. Cross-validation methods, including repeated stratified k-fold and leave-one-out cross-validation (LOOCV), confirmed model robustness. The final 11-biomarker model (e.g., SMlipids, kynurenine ratio) demonstrated an AUC of 98% and strong predictive capability [74]. A robust validation strategy increases confidence in the biomarker panel. However, this is still limited to a single-center cohort; future work should validate these findings in larger, independent cohorts.

### 4.3. Prognostic Modeling and Racial Disparities in Breast Cancer

Machine learning also facilitates the identification of prognostic markers and racial disparities in breast cancer outcomes. A GC-MS-based metabolomics study involved 250 participants, comprising 102 breast cancer patients (African American (AA) = 48; Non-Hispanic White (NHW) = 54) and 148 healthy controls (AA = 100; NHW = 48). Metabolomics data were preprocessed by removing metabolites with over 40% missing values, imputing remaining missing data, and conducting feature selection via Boruta and Recursive Feature Elimination (RFE). The Boruta algorithm identified 16 key metabolites. Machine learning classification used Decision Tree, Random Forest, Logistic Regression, and Support Vector Machine (SVM). Model evaluation was based on ROC curves, AUC values, Matthews Correlation Coefficient (MCC), and F1 score, with Random Forest combined with Boruta being the best-performing approach. Machine learning analysis employed a Random Forest classifier, achieving an AUC of 0.79 (MCC = 0.56) for AA individuals and an AUC of 0.78 (MCC = 0.52) for NHW individuals. Key metabolites like methionine (lower in AA) correlated with TCGA data showing elevated methyltransferase expression in AA tumors, suggesting epigenetic contributions to worse survival [8] (Tables S1 and S3). Nonetheless, plasma-based snapshots may inadequately reflect dynamic tumor evolution, and limited metabolite coverage (GC-MS: ~460 metabolites) could miss important lipids or polar compounds better captured via LC-MS.

A subsequent analysis integrated RNA-seq and metabolomics data to explore metabolite-driven signaling in Breast Invasive Carcinoma (BRCA) using a multi-task Gaussian process (GP) model. The workflow involved rigorous preprocessing (normalization and batch correction) and sparse variational multi-task GP (SVGP) with RBF/linear kernels to link 317 metabolites to signaling circuits (R^2^ > 0.5). Key metabolites like 2-aminoadipate and inosine were implicated in tumor aggressiveness and immune modulation, though their transient nature posed validation challenges. The GP model avoided overfitting, as shown by cross-validated R^2^ and MSE trends, and used SHAP for interpretability [75]. The limitations included sample bias and reliance on predicted rather than directly measured metabolite production. The findings not only highlight metabolites as potential therapeutic targets but also underscore the need for experimental validation of mechanistic predictions.

Across the reviewed studies, ML consistently demonstrated its capacity to harness metabolomics for BC subtyping, biomarker identification, treatment response prediction, and prognosis modeling. Unsupervised techniques such as SNF and PCA enabled initial stratification, while supervised approaches (e.g., LASSO, SVM, RF) provided high predictive accuracy (AUCs: 0.89–0.99). Particularly, SNF + SVM and DESI-MSI + LASSO pipelines effectively differentiated TNBC metabolic subtypes and tumor versus normal samples. Yet, interpretability was frequently constrained in models like SVM, and overreliance on linear classifiers (e.g., PLS-DA) in complex metabolic landscapes may reduce robustness.

In biomarker discovery, the integration of SHAP values added essential biological interpretability, particularly in treatment response settings (e.g., inetetamab). However, high training-validation performance gaps and the absence of external datasets limited translational readiness. Deep learning models and feature attribution frameworks remain underutilized but are essential for mechanistic insights.

Prognostic modeling efforts underscore the value of ML in uncovering racial disparities and molecular drivers of tumor aggression. RF models and GP frameworks successfully linked metabolic profiles to clinical phenotypes and signaling pathways. Nevertheless, limited sample sizes, platform constraints (e.g., GC-MS), and lack of experimental confirmation of model-predicted mechanisms persist as barriers.

## 5. Metabolomics-Based Machine Learning for Colorectal Cancer Detection

### 5.1. Colorectal Cancer Detection Using Metabolomics and Machine Learning

Colorectal cancer (CRC) detection has been enhanced by machine learning (ML) techniques applied to metabolomics data. One study utilized ML to classify metabolomics data related to CRC, focusing on selected metabolites and random compounds across different CRC stages. The dataset underwent statistical preprocessing, filtering out metabolites with *p*-values greater than 0.05. Feature selection using the InfoGainAttributeEval function reduced the number of attributes from 3679 to 937. Supervised ML algorithms were implemented in WEKA, with 10-fold cross-validation for evaluation. The best-performing models included Bagging classifiers for stages 0–2 (89.55% accuracy) and stages 0–4 (93.04% accuracy) and an AttributeSelectedClassifier for stages 3–4 (95.21% accuracy). Performance metrics such as accuracy, ROC-AUC, and precision–recall AUC were used to assess model reliability. Testing on an independent dataset confirmed the reliability of these classifiers in identifying CRC-related metabolites. Key metabolites like phenylalanine (associated with early stages) and arginine (associated with late stages) were linked to CRC pathways, suggesting their clinical utility for stage-specific diagnostics [76].

From a methodological standpoint, this study leveraged classical ensemble-based algorithms known for their robustness to overfitting and high-dimensional data. However, the dataset’s small size and limited hyperparameter optimization present barriers to generalizability. Moreover, the classifiers’ diminished performance on unrelated cancer types, such as thyroid cancer (65.22% accuracy), underscores the specificity limitations of the trained models. The authors correctly acknowledged the need for expanded training sets and further exploration of metabolite diversity to improve performance across heterogeneous datasets. The study demonstrates that the selection of feature reduction strategies and classifier types can significantly impact stage-specific detection, yet it also emphasizes the importance of generalizability and external validation (Tables S1 and S3).

A separate investigation aimed to develop ML models for CRC screening using metabolomics data from 76 stool-derived microbial EV samples (36 CRC patients; 40 healthy controls). The workflow included GC/LC-TOFMS data generation, univariate/multivariate statistical analysis, and supervised ML (SVM, PLS-DA, and RF) to classify CRC vs. healthy controls. Five metabolites were found to be significant, with SVM outperforming others (AUC 0.985). Succinic acid and branched-chain amino acids (leucine and isoleucine) were upregulated, aligning with prior CRC metabolomics studies, while butyric acid downregulation suggests gut microbiome dysbiosis. A 10-fold cross-validation strategy validated the model’s robustness [7]. Despite the small sample size, the study surpassed the MetSizeR minimum estimate and underscored microbial EVs’ diagnostic potential. However, the absence of external validation and omission of multi-omics integration limit mechanistic insight and translational readiness.

### 5.2. Multi-Omics Approaches for Colorectal Cancer Prediction

Advancements in predictive modeling have integrated multi-omics data, including microbiome and metabolomics analyses. A large-scale study investigated human gut microbiota samples from 2951 individuals, including healthy, colorectal adenoma (CRA), and CRC patients. Five omics data types (taxonomy, functional genes, enzymatic reactions, metabolic pathways, and predicted metabolites) were processed using a custom pipeline (MetaBakery). Data preprocessing involved feature selection using the web-based machine learning platform “Just Add Data Bio” (JADBIO), Mann–Whitney U statistical tests, and filtering rows with at least 1250 non-zero entries. Random Forest models, optimized for each dataset, showed the highest AUC scores in taxonomy (train: 0.817; test: 0.787), functional genes (train: 0.815; test: 0.822), enzymatic reactions (train: 0.825; test: 0.812), and metabolic pathways (train: 0.799; test: 0.768). Predicted metabolites performed worst (train: 0.621; test: 0.606). Unsupervised learning through Linear Discriminant Analysis (LDA) and Uniform Manifold Approximation and Projection (UMAP) helped visualize group clustering, with LDA effectively distinguishing CRC-related groups except for predicted metabolites. A final integrated model incorporating key features achieved an AUC of 0.87, demonstrating enhanced predictive accuracy [77].

The study’s clinical relevance lies in its multi-omics approach, highlighting microbial functional shifts in CRC progression. However, limitations include the lack of external validation and low predictive power of metabolites, likely due to reliance on in silico predictions (MelonnPan) rather than empirical metabolomics. The 70:30 internal validation split may also overestimate performance. Future work should prioritize empirical quantification of metabolites and larger, diverse cohorts to improve generalizability.

### 5.3. Alternative Biological Fluids for Colorectal Cancer Detection

The exploration of alternative biological fluids for CRC detection has led to promising findings in salivary and serum metabolomics. A study utilizing unstimulated saliva samples from 2602 individuals (235 CRC, 50 Alzheimer’s disease (AD), and 2317 healthy controls) aimed to identify CRC biomarkers. The dataset was split into training (*n* = 1301) and validation (*n* = 1301) sets. Statistical analysis (Mann–Whitney U test) identified 63 significant metabolites, with 23 showing fold changes greater than 1.71, leading to feature selection using Variable Importance in Projection (VIP) scores. Among these, N-acetylputrescine emerged as a key biomarker. Supervised ML models, including Alternative Decision Tree (ADTree) and Multiple Logistic Regression (MLR), were trained. ADTree models achieved the highest performance (AUC: 0.933–0.951 in training, 0.870 in validation), significantly outperforming MLR models (*p* < 0.0001). Unsupervised analysis via PLS-DA effectively separated CRC from non-CRC groups. Sensitivity comparisons with tumor markers showed that ADTree and MLR models provided better classification performance. The findings suggest that salivary acetylated polyamines reflect CRC-associated metabolic dysregulation (e.g., Warburg effect, MYC-driven ODC activation). Clinically, this approach offers a non-invasive screening tool with 77% sensitivity for CRC and 68% for AD [11]. Despite promising performance, specificity for distinguishing cancer from other chronic diseases remains a limitation (Tables S1 and S3).

In another study, serum metabolomics data from 200 individuals (healthy subjects (HS), benign colorectal tumors (BCRT), and CRC) were analyzed using Gas Chromatography–Mass Spectrometry (GC-MS). A cohort of 200 subjects (50 HS, 50 BCRT, and 100 CRC) was split into training (*n* = 133) and test (*n* = 67) sets. Data preprocessing included log transformation, autoscaling, batch correction, statistical analysis, feature selection through a genetic algorithm, and metabolite-set enrichment analysis. Ten supervised ML models, including Naïve Bayes (NB), Generalized Linear Model (GLM), Logistic Regression (LR), Flexible Linear Modeling (FLM), Deep Learning (DL), Decision Tree (DT), Random Forest (RF), Gradient Boosting Tree (GBT), SVM, and PLS-DA, were trained to distinguish CRC from controls. The best-performing model was an ensemble model (EML), achieving 100% accuracy. Feature selection identified 12 key metabolites crucial for classification. The optimal PLS-DA model used four latent components, yielding R2 = 0.907 and Q2 = 0.787. Key metabolites aligned with known cancer metabolism (Warburg effect and glutamine addiction) and microbiome-derived SCFAs. The limitations include single-center bias and lack of external validation [12]. The study highlights the potential of metabolomics–ML integration for non-invasive CRC screening but warrants multicenter validation to confirm robustness.

These studies illustrate that biofluid choice, analytical platform (e.g., GC-MS vs. LC-MS), and model complexity (e.g., ensemble vs. linear regression) significantly impact diagnostic performance. While salivary and serum-based diagnostics offer high accuracy and clinical feasibility, rigorous validation remains essential (Tables S1 and S3).

Across the reviewed studies, machine learning-driven metabolomics has emerged as a powerful tool for CRC detection and classification. Early-stage studies demonstrated that classical ensemble methods (e.g., Bagging, AttributeSelectedClassifier) could accurately differentiate CRC stages (accuracy up to 95.2%) yet suffered from limited generalizability to unrelated conditions. In contrast, SVM outperformed other models in microbial EV-based studies (AUC = 0.985), showcasing its strength in small, well-curated datasets.

The multi-omics study demonstrated the value of integrating diverse biological layers, with microbial enzymatic reactions and functional gene profiles achieving the highest predictive power (AUCs > 0.8). However, reliance on in silico-predicted metabolites reduced the relative contribution of metabolomics in this framework. Future efforts must prioritize experimentally derived metabolite data to preserve biological fidelity.

Alternative fluids such as saliva and serum yielded promising diagnostic models. Salivary ADTree classifiers performed well (AUC up to 0.951), while serum-based ensemble models reached 100% accuracy. However, single-center study designs and the absence of validation in independent cohorts limit translational potential. These models highlight the importance of balancing model complexity, biological interpretability, and clinical feasibility.

## 6. Machine Learning Approaches for Ovarian Cancer Diagnosis Using Metabolomics

### 6.1. Plasma and Serum Metabolomics for Ovarian Cancer Classification

In one study, metabolomics profiling was applied to plasma extracellular vesicles (EVs) to identify ovarian cancer biomarkers in 105 participants (37 OC, 22 BE, and 46 CON). Data preprocessing included normalization, batch correction, and RF-RFE for feature selection. Metabolite profiling revealed significant alterations in 19 and 158 metabolites in ovarian cancer (OC) vs. benign ovarian tumor (BE) and OC vs. control patients (CON) comparisons, respectively.

Feature selection reduced 388 features to 12 key metabolites. UMAP-based dimensionality reduction showed distinct separation between groups. Several supervised machine learning algorithms were employed for binary classification, with random forest (RF) as the top performer (AUC = 0.91; F1 = 0.88), artificial neural network (ANN) (AUC = 0.90, F1 = 0.83), and SVM (AUC = 0.94 for OC-BE) achieving the best results. The dataset was split into training (60%), testing (20%), and validation (20%) to prevent overfitting. Key metabolites like 4-morpholineethanamine and maltol were implicated in cancer pathways, though their novelty limits direct clinical translation. The study highlights the potential of metabolic biomarkers in plasma EVs for ovarian cancer diagnosis [10].

In another study, a machine learning (ML) classifier was developed using serum metabolomics data to distinguish ovarian cancer (OC) patients from noncancerous individuals. A total of 564 serum samples (431 OC and 133 controls) were analyzed using UPLC-MS/MS with HILIC and C18 reversed-phase chromatography, resulting in four datasets (positive HILIC (HN), negative HILIC (HP), reversed-phase negative (RN), reversed-phase positive (RP)). Quality control was performed using PCA to confirm no batch effects. Feature selection was conducted using Recursive Feature Elimination (RFE) with cross-validation, ranking features based on frequency and Gini importance scores. A neural network-based autoencoder was applied for dimensionality reduction before ML analysis. Five supervised ML classifiers—Random Forest (RFC), Support Vector Classifier (SVC), Adaptive Boosting (ADA), K-Nearest Neighbors (KNN), and Logistic Regression Classifier (LRC)—were independently evaluated. Performance metrics included Positive Predictive Value (PPV ≥ 93%), Negative Predictive Value (NPV ≥ 87%), F1 score (≥92%), and Matthews Correlation Coefficient (MCC ≥ 0.78). The classifiers demonstrated high accuracy across all datasets, with consensus models further enhancing prediction. Stratified k-fold cross-validation validated results. Early-stage cancers were classified with 98% accuracy, and late-stage cancers with 92.7%. Clinically, probabilistic scoring proposed a framework for personalized diagnostics, though prospective validation is needed [64] (Tables S1 and S3).

The key limitations included reliance on unannotated metabolites and dataset-specific biases. Future work should expand sample diversity and integrate multi-omics data to improve generalizability

### 6.2. Multi-Sample Metabolomics Analysis in Ovarian Cancer

In this study, the authors employed GC-MS-based metabolomics to profile 96–130 metabolites across plasma, urine, hair, and ovarian tissue in OC, BOT, and control groups. Data preprocessing included log2/z-score normalization, batch correction, and RFE for feature selection. PCA was used for unsupervised metabolite comparison, while seven supervised ML models were trained, with SVM and NB achieving perfect AUCs (1.0) in tissue/urine. Key metabolites (e.g., succinic acid, glutamic acid) were linked to energy metabolism and ferroptosis, highlighting their potential as OC biomarkers. External validation confirmed robustness (AUC > 0.8). Clinically, urine metabolites showed high diagnostic potential (AUC > 0.95), suggesting non-invasive screening utility. Elevated TCA cycle intermediates and amino acids in OC tissues align with Warburg effect theories [65].

## 7. Bladder Cancer Prognostic and Diagnostic Models

One study leveraged integrative ML to develop a Nicotine-Related Signature (NRS) for bladder cancer prognosis. Unsupervised clustering of 63 NRGs revealed three patient clusters with distinct survival outcomes, linking nicotine metabolism to tumor microenvironment (TME) heterogeneity. Supervised ML (Random Survival Forest) identified a four-gene NRS (AHNAK, MKRN1, SFXN4, and ZMYND8) with robust prognostic power (C-index = 0.763; AUC > 0.97). The NRS stratified patients into high-/low-risk groups, where high-risk patients exhibited worse OS, elevated immune checkpoint expression (PD-L1 and CTLA4), and CAF-rich TME—features associated with immunotherapy resistance. Functional analyses implicated cytokine/PI3K-Akt pathways in NRS-mediated progression. Experimental validation confirmed MKRN1’s oncogenic role, with nicotine exacerbating BLCA progression via MKRN1 [78]. This study underscores the role of nicotine metabolism genes in BLCA prognosis and the potential application of machine learning in clinical oncology.

Another study involved 152 subjects (87 bladder cancer patients and 65 hernia patients), with urine samples analyzed using UPLC-TOF-MS for metabolomic profiling. The dataset was split into a training set (105 samples) and a testing set (47 samples). Preprocessing included statistical analysis (showing hemoglobin was significantly lower in bladder cancer patients), feature selection (identifying six metabolite markers via a pipeline using Wilcoxon tests, fold-change filtering, and AUC thresholds), and clustering validation using Locally Linear Embedding (LLE). A decision tree model was trained using 5-fold cross-validation, achieving an accuracy of 84.76%, sensitivity of 81.82%, and specificity of 88.00%. When tested on the independent dataset, the model achieved an accuracy of 76.60%, sensitivity of 71.88%, and specificity of 86.67%. Unsupervised clustering (LLE) confirmed the discriminative power of selected features. The sole annotated metabolite, imidazoleacetic acid, aligns with histamine-driven inflammation pathways implicated in BCa progression [79] (Tables S1 and S3).

Together, these studies illustrate the versatility of ML in tackling both prognostic and diagnostic challenges in bladder cancer, though with different methodological emphases and clinical endpoints. The gene expression-based prognostic model demonstrated high predictive performance (AUC > 0.97) and deep biological integration, connecting nicotine metabolism, immune evasion, and disease progression. However, its reliance on tumor transcriptomic data limits practical implementation in routine clinical settings due to the invasiveness of tissue sampling and the complexity of RNA sequencing.

In contrast, the urine-based diagnostic model offers a non-invasive and accessible alternative, showing moderate-to-high performance in distinguishing BCa from benign conditions using a simple decision tree and a small number of features. While less comprehensive mechanistically, its potential for early detection and screening is promising, especially if biomarker annotation and classifier optimization are improved.

## 8. Machine Learning Approaches for Lung Cancer Diagnosis, Subtyping, and Treatment Response Using Metabolomics

### 8.1. Metabolomics and Machine Learning in Lung Cancer Diagnosis and Subtyping

One study leveraged LC-MS/MS-based metabolomics data from 461 serum samples to distinguish SCLC, NSCLC, and healthy controls using a stacking ensemble approach. Preprocessing included StandardScaler normalization and feature selection. Feature selection was performed using XGBoost for multi-class classification and Extra Trees for binary classification, identifying the top 47 and 48 features, respectively.

Multi-class classification models included CatBoost, SVM, MLPClassifier, Extra Trees, GradientBoosting, and XGBoost. Tree-based models (CatBoost and RandomForest) excelled in multi-class tasks, while MLPClassifier and SVM performed best in binary classification. The ensemble improved AUC to 0.92–0.95, with SHAP analysis highlighting key metabolites like benzoic acid and DL-lactate. Five-fold cross-validation was used for model evaluation, ensuring robustness. AUC-ROC analysis further validated the models, confirming their ability to distinguish between cancer subtypes effectively [9].

A conceptually distinct yet complementary approach employed a two-tiered neural network to classify four lung cancer subtypes (adenocarcinoma (AD), squamous cell carcinoma (SQ), non-small-cell lung cancer (NS), and small-cell carcinoma lung (SC)) using serum metabolomics data. The workflow began with stringent filtering (*p* < 0.05, FC ≥ 1.2) and conversion of SMILES to PaDEL descriptors, followed by InfoGain-based feature selection to reduce noise. Dimensionality reduction was tailored to data linearity: t-SNE for NS/SC (nonlinear) and PCA for AD/SQ (linear). A three-layer DNN achieved high accuracy (92% with t-SNE), which was validated via cross-validation and independent datasets. Pathway analysis revealed cancer-specific metabolic disruptions (e.g., ketone bodies in NS, glutathione in AD), underscoring the model’s biological plausibility [80]. However, the limitations include the following: Small training sets (35–46 metabolites/subtype) may limit generalizability, InfoGain may overlook synergistic feature interactions, lack of original platform details complicates reproducibility, and the errors in the first tier (NS/SC) propagate to AD/SQ. Despite this, the study demonstrates the utility of metabolite-based ML classifiers in oncology, with t-SNE emerging as superior for nonlinear metabolomic data (Tables S1 and S3).

### 8.2. Metabolomics Biomarkers for Lung Cancer Detection and Treatment Response

Other studies focused on identifying specific metabolomics biomarkers for lung cancer detection and evaluating treatment responses. One investigation integrated metabolomics and machine learning to identify salivary biomarkers for lung cancer detection. Using CE-TOF-MS and LC-QQQ-MS, the team analyzed 109 tissue, 71 plasma, and 83 saliva samples, identifying 12 metabolites (e.g., acetylated polyamines, amino acids) consistently altered across sample types. N1-acetylspermidine emerged as the top single-metabolite discriminator (AUC = 0.758), while an ADTree model combining multiple metabolites achieved a higher AUC (0.792). The workflow included rigorous statistical testing (FDR-corrected *p* < 0.05), stepwise feature selection, and cross-validation, demonstrating robustness against confounders like age or smoking status. Clinically, elevated salivary acetylated polyamines reflect tumor metabolic reprogramming (e.g., MYC-driven polyamine synthesis), offering a non-invasive diagnostic avenue. However, limitations include an unmatched age between cases/controls and a lack of external validation. Future work should address oral microbiome influences and develop point-of-care assays [81].

In a different study, metabolomics data from serum samples of NSCLC patients and healthy controls were integrated to evaluate pemetrexed treatment efficiency. Discovery (*n* = 122) and validation (*n* = 201) cohorts revealed tryptophan metabolism (kynurenine and xanthurenic acid) as key discriminators. Preprocessing included IS normalization, PCA/PLS-DA, and FDR-corrected ANOVA. Eight ML algorithms were evaluated, with RF achieving a superior AUC (0.98 for healthy (H) vs. non-disease (ND); 0.95 for sensitive (S) vs. resistant (R)). The RF model’s decision tree highlighted KTR and XKR as critical ratios for clinical stratification, outperforming traditional biomarkers (neutrophils/lymphocytes). Clinically, tryptophan metabolites may serve as non-invasive biomarkers for pemetrexed efficacy, aiding personalized therapy [82] (Tables S1 and S3).

The four studies reviewed provide complementary insights into the capabilities and current challenges of integrating metabolomics with machine learning for lung cancer applications. The diagnostic and subtyping studies emphasize model architecture and feature handling as key determinants of performance. Ensemble and tree-based models, particularly when combined with robust feature selection (e.g., XGBoost, Extra Trees), proved effective for multi-class classification, while the neural network approach demonstrated strengths in subtype-level granularity, particularly when paired with dimensionality reduction strategies such as t-SNE.

In terms of diagnostic biomarkers, the use of multiple biospecimens—especially saliva—offers promise for non-invasive screening, while serum-based profiling remains more established for clinical applications. Salivary metabolite signatures (e.g., N1-acetylspermidine) yielded moderate classification performance but may support low-cost, rapid diagnostics when validated externally. In contrast, serum biomarkers associated with tryptophan metabolism demonstrated superior performance in predicting treatment response, highlighting their potential for guiding personalized therapy. Notably, both diagnostic and therapeutic models employed interpretable algorithms (ADTree, Random Forest), enhancing their translational viability.

Methodologically, the reliance on different feature selection strategies (e.g., InfoGain vs. XGBoost) and dimensionality reduction techniques reflects varying trade-offs between speed, interaction modeling, and interpretability. From an expert perspective, future pipelines should incorporate multi-modal omics data, deploy explainable AI tools (e.g., SHAP, LIME), and ensure model reproducibility through platform transparency and external validation. Hierarchical models, though powerful, require strategies to mitigate error propagation, and nonlinear embeddings like t-SNE may offer critical advantages in subtype separation.

Ultimately, integrating these approaches into cohesive, dual-purpose pipelines—capable of both early detection and treatment decision-making—represents the next frontier. Such frameworks would benefit from the temporal modeling of metabolite trajectories, the inclusion of microbiome interactions, and the development of clinically deployable assays. These refinements are essential for advancing ML–metabolomics tools from proof of concept to practice-changing clinical applications in lung cancer management.

## 9. Metabolomics and Machine Learning in Diagnosing Pancreatic and Related Diseases

Metabolomics data combined with machine learning has shown remarkable potential in diagnosing pancreatic cancer and differentiating it from other diseases. One study leveraged LC/ESI-MS-based metabolomics and machine learning to develop a diagnostic model for pancreatic ductal adenocarcinoma (PDAC) using serum samples. The workflow included rigorous preprocessing (median normalization and autoscaling) and feature selection. Thirty-six significant primary metabolites (PMs)/phospholipids (PLs) were identified through statistical filtering (*p* < 0.03, log2 fold-change > 0.6). Partial Least Squares Regression (PLSR) was applied for initial clustering, while SVM achieved high accuracy (AUC = 0.9974), distinguishing PDAC from controls, and it was validated in an independent cohort (AUC = 0.99). Key metabolites like ornithine and phosphatidylethanolamine (PE) (38:5) were upregulated in PDAC, while sphingomyelin (SM) (36:4) and phosphatidylcholine (PC) (44:2) were downregulated, implicating disrupted lipid metabolism and arginine biosynthesis.

Neoadjuvant chemotherapy (NAC) therapy partially reversed PDAC-associated metabolic changes, positioning NAC samples between control and untreated PDAC in the Partial Least Squares Regression (PLSR) space. The limitations include a small sample size (*n* = 29 PDAC cases), lack of external validation cohorts, and unspecified SVM hyperparameters, which may affect reproducibility. The study also did not address potential confounding factors like diet or comorbidities in controls [15].

From a machine learning perspective, the use of SVM—a robust kernel-based classifier—is appropriate for small, high-dimensional datasets. However, the lack of transparency around hyperparameter optimization and the small sample size (*n* = 29 PDAC) limit reproducibility. Moreover, potential confounders like comorbidities and diet were not accounted for, which may affect the model’s generalizability in clinical settings.

Another study focused on distinguishing immunoglobulin G4-related disease (IgG4-RD) from pancreatic cancer (PC), Sjogren’s syndrome (SS), and healthy controls (HCs). Plasma samples from 151 patients (87 with IgG4-RD, 33 with PC, 31 with SS, and 30 HCs) underwent untargeted metabolomics analysis. Preprocessing included sum normalization and Pareto scaling, followed by PCA/OPLS-DA for exploratory analysis and Random Forest for biomarker discovery. PCA and OPLS-DA confirmed model reliability (R2 and Q2 values), while random forest selected key metabolites and served as a supervised learning classifier. ROC analysis demonstrated exceptional diagnostic performance, with AUC values of 1 for distinguishing IgG4-RD from HC, PC, and SS. Five essential metabolic biomarkers (e.g., caftaric acid, hydroxyproline) were identified, underscoring the power of machine learning in enhancing metabolomics-based disease classification and differentiation. Clinically, these metabolites correlated with IgG4 levels and inflammatory markers, suggesting roles in disease mechanisms (e.g., ABC transporters) [83] (Tables S1 and S3).

Both studies highlight the ability of metabolomics-driven machine learning approaches to achieve high diagnostic accuracy, especially in pancreatic diseases and related conditions.

Across both studies, SVM and Random Forest emerged as powerful classifiers for metabolomics-based disease differentiation. The PDAC-focused study leveraged SVM with feature selection and dimensionality reduction to achieve extremely high diagnostic accuracy (AUC = 0.9974), although its limited sample size and lack of external validation present challenges for generalizability. In contrast, the second study applied Random Forest to a larger and more diverse cohort, achieving perfect classification (AUC = 1) for distinguishing IgG4-RD from multiple conditions, including pancreatic cancer—demonstrating excellent robustness across disease classes.

While the first study emphasized lipid and amino acid metabolism disruptions as PDAC markers, the second identified inflammation-related metabolites linked to IgG4-RD pathogenesis. Notably, the PDAC model also attempted to track treatment response via NAC sample profiling, offering early steps toward dynamic treatment monitoring. However, this approach lacked validation and interpretability, partly due to unspecified model parameters and potential confounders like diet.

## 10. Metabolomics-Based Machine Learning for Thyroid Cancer Diagnosis

Metabolomics data, paired with machine learning, has shown significant promise in improving thyroid cancer diagnostics. One study developed a machine learning system using a dataset of 128 metabolites, refined to 53 attributes after filtering. Among various classifiers tested, LogitBoost achieved the highest accuracy of 87.03%. The model was validated using ten-fold cross-validation, with performance metrics such as ROC curves, AUC, and precision–recall curves confirming its robustness. When tested on kidney disease metabolites, the system produced a 7% classification rate, whereas it achieved 100% accuracy when tested on thyroid cancer serum metabolites, demonstrating its potential in identifying thyroid cancer-specific metabolic patterns [14].

Similarly, another study analyzed fine-needle aspiration cytology (FNAC) metabolomic data from 78 patients with thyroid nodules (TNs): 26 benign TNs (Bethesda II), 30 benign and 11 papillary thyroid carcinomas (PTCs) classified as indeterminate (Bethesda III–IV), and 11 PTCs categorized as suspicious/malignant (Bethesda V–VI). The data underwent feature selection through PLS-DA, reducing the set to 15 key metabolites. Several supervised algorithms were tested, with a supervised autoencoder (SAE) achieving the highest accuracy (0.957) and AUC (0.945). PLS-DA, Random Forest (RF), and Support Vector Machines (SVMs) also demonstrated strong classification performance, though unsupervised PCA failed to separate benign from malignant samples. SAE maintained high performance across 12-fold cross-validation, while SVM and RF were validated with fourfold cross-validation. Permutation tests further confirmed the robustness of PLS-DA [84]. These findings collectively highlight the potential of metabolomics-driven machine learning for accurate, reliable thyroid nodule classification and thyroid cancer diagnostics.

These two studies illustrate the complementary aspects of metabolomics–ML integration for thyroid cancer diagnostics. The serum-based LogitBoost model offers a non-invasive, systemic overview useful for screening or surveillance, while the FNAC-based SAE model directly targets diagnostic ambiguity in indeterminate nodules, a longstanding clinical challenge.

From a machine learning perspective, the contrast between LogitBoost and SAE highlights how model selection and dataset structure shape predictive performance. LogitBoost is well-suited for binary classification with moderately sized datasets, while SAE’s capacity for feature compression and nonlinear pattern recognition offers superior performance in complex, multi-class settings, such as Bethesda categories.

However, several challenges remain. Both studies rely on relatively small, single-center cohorts, limiting generalizability. Furthermore, feature annotation and pathway interpretation were limited, constraining biological validation. In particular, the metabolite signatures identified in FNAC samples require further exploration to establish mechanistic links to thyroid carcinogenesis. Despite these limitations, both studies underscore the diagnostic potential of metabolomics-based machine learning models for thyroid cancer.

## 11. Metabolomics and Machine Learning for Prostate Cancer Diagnosis and Classification

Metabolomics data combined with machine learning has shown promising results in prostate cancer (PCa) diagnosis and classification. One study analyzed metabolomics data from tumor tissue, normal tissue, and serum samples to classify tumor versus normal tissue and differentiate high versus low Gleason scores. The dataset included 33 high- and 53 low-Gleason-score tumor samples, along with serum data from two cohorts. Automated ML (AutoML), which consists of two phases, Bayesian hyperparameter optimization followed by greedy ensemble construction, emerged as the top-performing supervised model, achieving a cross-validation AUC of 0.82 ± 0.08 and a test-set AUC of 0.79 for tumor versus normal classification. However, the model struggled to differentiate high versus low Gleason scores, with an AUC of 0.67 for tissue and 0.56 for serum. Unsupervised clustering successfully distinguished tumor from normal samples but failed to separate high from low Gleason grades. Feature importance analysis identified key metabolites such as 3-phosphoglycerate, N-acetylputrescine, ATP, glutathione (GSH), and nicotinamide—findings that aligned with univariate statistical analysis. The study suggests that while strong metabolic signals for differentiating high-grade from low-grade cancer are lacking, metabolomics-based machine learning remains effective for tumor detection [13].

In another study, UPLC-MS metabolomics data from serum samples of PCa patients and healthy individuals were analyzed to develop a diagnostic model. Data preprocessing involved hypothesis testing (*p*-value = 0.0099) and recursive feature elimination (RFE), reducing the initial 480 features to an optimal subset of 40. A supervised Support Vector Machine (SVM) classifier, trained and tested over 10 iterations, achieved impressive performance metrics: 93.0% accuracy, 92.1% sensitivity, and 94.3% specificity. Leave-one-out cross-validation (LOOCV) did not outperform the original model, suggesting the original pipeline’s robustness. To further ensure reliability and rule out overfitting, Principal Component Analysis (PCA) was applied, showing clear class separation with the reduced 40-feature subset (33.6% variance explained by 3 components), while the original feature set failed to produce similar separation. This highlights the effectiveness of the RFE-SVM approach in isolating key metabolic signatures for accurate PCa detection [85].

Across both studies, SVM emerged as a robust classifier—particularly when paired with rigorous feature selection techniques such as RFE. Meanwhile, AutoML provided flexibility in model exploration, but the Gleason-grade prediction failures suggest that biological signal limitations—not model architecture—were likely the primary constraint. Moreover, the failure of LOOCV to outperform the baseline in the second study implies that overfitting risk can be mitigated with thoughtful pipeline design, even in relatively small datasets.

Both studies demonstrate a high diagnostic utility for distinguishing tumor from non-tumor samples yet underscore a recurring challenge in PCa metabolomics: detecting grade-dependent metabolic changes remains elusive, likely due to intra-tumoral heterogeneity and overlapping metabolic signatures. Additionally, serum-derived models may be less sensitive to localized tumor grading compared to tissue-based analyses, though they are more amenable to clinical applications due to their non-invasive nature.

To advance the clinical translation of ML-driven metabolomics across cancer types, several overarching strategies should be prioritized. Integrating multi-omics data—such as transcriptomics, proteomics, spatial imaging, and microbiome profiles—can enhance the biological interpretability of models and uncover robust, clinically relevant biomarkers. The use of explainable AI tools like SHAP and LIME is essential to improve transparency, build clinician trust, and clarify the biological contribution of selected features. Expanding sample size and diversity through multi-center collaborations and incorporating longitudinal sampling can improve model generalizability, stability, and relevance across heterogeneous patient populations. External and prospective validation remains critical to ensure reproducibility and mitigate overfitting. Furthermore, employing multi-class modeling and combining non-invasive metabolite signatures with genomic or immunophenotypic data can better capture disease complexity and support early detection, subtype classification, and personalized treatment planning. Together, these strategies offer a path forward for refining ML–metabolomics pipelines into clinically actionable tools in precision oncology.

## 12. Limitations, Challenges, and Future Perspectives of Integrating Machine Learning (ML) with Cancer Metabolomics

### 12.1. Data Quality and Preprocessing Challenges: The Foundation That Shapes All Outcomes

Among the foundational challenges in ML–metabolomics is the issue of data quality—particularly the prevalence of missing values, batch effects, and inconsistencies in normalization methods. In our view, this represents the most fundamental and pervasive barrier, as it directly compromises the input layer of ML models and subsequently cascades into every aspect of downstream analysis. We consider the issue of data quality and preprocessing to be the most foundational challenge, as it directly affects the reliability of all downstream ML modeling efforts. Metabolomics datasets often suffer from high rates of missingness due to technical variability or low-abundance metabolites. Although imputation techniques such as k-nearest neighbors (k-NNs) and multiple imputation by chained equations (MICEs) are frequently used, they may introduce bias and reduce model reliability, particularly when applied without consideration of biological context [86]. In addition, batch effects stemming from variations in sample preparation or instrument performance can obscure true biological signals. Tools like ComBat and Surrogate Variable Analysis (SVA) provide partial correction but may not fully eliminate these effects, especially in large multi-center studies [87,88]. Inconsistent normalization and scaling methods can lead to biased results. For example, Total Ion Current (TIC) normalization may not account for all technical variability, while Z-score normalization assumes a normal distribution, which may not hold for all metabolites [89].

Future Directions: To improve robustness and reproducibility, we advocate for the development of standardized preprocessing pipelines tailored specifically to metabolomics. Deep-learning-based imputation tools, such as autoencoders or variational models, should be explored to handle missing data while preserving biological structure. Probabilistic Quotient Normalization (PQN) and other biologically informed scaling methods offer more stable normalization and should be validated across diverse datasets. Establishing a minimal preprocessing standard across metabolomics studies would dramatically enhance data harmonization and model reproducibility.

### 12.2. Model Overfitting and Generalizability: A Barrier to Translation

Model overfitting remains a critical barrier to deploying ML-based metabolomics tools in clinical settings. This is particularly problematic in studies with small sample sizes, such as those with fewer than 30 cancer patients, where models may perform well in internal cross-validation but fail in external validation [90].

Several studies lack external validation cohorts, relying solely on cross-validation. Without validation in independent datasets, the predictive performance of ML models may not hold in real-world clinical settings [91]. Class imbalance, where one class (e.g., healthy controls) dominates, can also lead to biased model training and inflated performance metrics. Although common strategies like synthetic minority oversampling (SMOTE) or propensity score matching are used to address imbalance, they do not always resolve the issue, particularly when sample heterogeneity is high [92].

Future Directions: The generalizability problem can only be solved through a combination of methodological innovation and larger, more representative datasets. We believe that multi-center collaborations and the reuse of large public resources such as The Cancer Genome Atlas (TCGA) or METABRIC are essential to increase sample diversity and scale. Transfer learning and ensemble modeling can help stabilize performance across studies and disease subtypes. Critically, we recommend that external validation using independent cohorts become a standard requirement for publishing ML–metabolomics models with clinical aspirations.

### 12.3. Interpretability and Biological Relevance: Bridging Prediction and Insight

While accuracy is a key strength of many ML models, interpretability is often lacking—particularly with black-box methods such as deep neural networks and random forests. This compromises clinical trust and limits biological insight. In our view, this issue is not purely technical but reflects a broader misalignment between computational modeling and biomedical needs [62]. Furthermore, many studies identify statistically significant metabolic features without confirming their biological relevance through experimental validation. This gap restricts the translational value of the findings and hampers integration into clinical decision-making [93].

Future Perspectives: To foster interpretability, researchers should adopt explainable AI (XAI) methods such as SHAP (SHapley Additive exPlanations) and LIMEs (Local Interpretable Model-Agnostic Explanations) during model development rather than as post hoc add-ons. Whenever possible, simpler and inherently interpretable models (e.g., rule-based learners, decision trees) should be used during early feature selection [62]. Importantly, experimental validation of ML-identified biomarkers should become an integral part of the discovery pipeline—through targeted metabolomics assays, pathway analysis, and functional perturbation studies.

### 12.4. Multi-Omics Integration: A Valuable Yet Underutilized Approach

Integrating metabolomics with other omics data—such as genomics, transcriptomics, and proteomics—offers a systems-level view of cancer biology. Yet, in practice, multi-omics integration remains rare due to differences in data types, dimensionality, and scale. Computational complexity and the lack of interoperable software platforms make integration a steep barrier, especially for non-computational labs [94]. Multi-omics integration increases computational complexity, requiring high-performance computing resources and advanced algorithms, which may not be accessible to all researchers [95].

Future Directions: We see strong potential in tools such as DIABLO, MOFA, and network-based integration frameworks, which enable dimensionality reduction and correlation analysis across omics layers. However, their wider adoption requires accessible, cloud-based platforms and training modules aimed at biologists. FAIR-compliant data standards and metadata harmonization should be promoted to enable seamless integration. We encourage journals and funding agencies to support integrated, cross-omics projects with interdisciplinary teams.

### 12.5. Racial and Ethnic Disparities: A Scientific and Ethical Priority

Many studies, such as those investigating metabolic signatures in African American and Non-Hispanic White breast cancer patients, are limited by the lack of diversity in their cohorts. This limits the generalizability of identified biomarkers and risks embedding bias into future clinical tools to other racial and ethnic groups [8].

Future Directions: In our opinion, addressing population diversity is not just a social responsibility but a scientific necessity. Future studies must incorporate inclusive recruitment strategies and explicitly report demographic composition, prioritize inclusive study designs that capture the metabolic diversity across racial and ethnic groups, and use diverse cohorts to identify biomarkers and therapeutic targets that are effective across populations. Federated learning and domain adaptation techniques may help develop models that generalize across diverse cohorts without requiring raw data sharing. Moreover, diversity-sensitive stratification in analysis pipelines should be encouraged to identify both population-specific and cross-ethnic biomarkers.

### 12.6. Clinical Translation: From Promise to Practice

A striking gap persists between ML–metabolomics discoveries and their real-world clinical use. Few models have been evaluated in prospective clinical trials, and even fewer have achieved regulatory approval. The lack of point-of-care technologies also hinders real-time decision-making.

**Figure 2:**
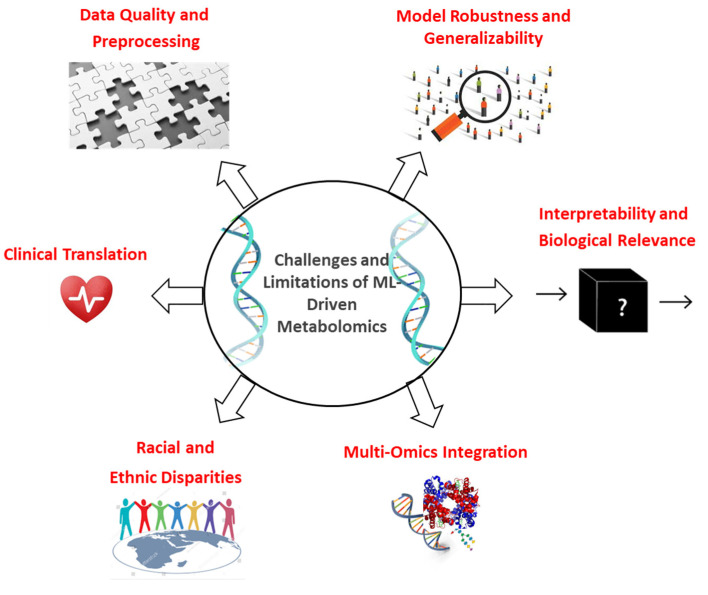
From bench to bedside: addressing challenges in machine learning-driven metabolomics for cancer.

Future Directions: Bridging this gap will require a concerted focus on translational infrastructure. ML-derived biomarkers should be validated in multi-center prospective trials, ideally embedded within ongoing clinical workflows. Simultaneously, portable, cost-effective devices—such as miniaturized mass spectrometers and biosensors—must be developed for bedside metabolomics analysis (Figure 2). Engaging with regulatory agencies early in the development process can help ensure that clinical-grade ML models meet the necessary standards for approval and integration into electronic health records.

Among the various limitations discussed, we view data quality and model generalizability as the most critical obstacles. Without addressing these, even the most sophisticated models will remain scientifically and clinically fragile. Interpretability, inclusion, and integration are equally pivotal to ensure that ML–metabolomics is not only effective but also ethical, accessible, and applicable. Overcoming these challenges will require not only technical solutions but also cultural shifts in how studies are designed, validated, and interpreted across the field.

## 13. Conclusions

The integration of machine learning with metabolomics has revolutionized cancer research, enabling the identification of metabolic subtypes, the discovery of diagnostic and prognostic biomarkers, and the development of predictive models. However, challenges related to data quality, model generalizability, interpretability, and clinical translation must be addressed to fully realize the potential of this approach. By improving data standardization, enhancing model robustness, advancing multi-omics integration, and addressing racial disparities, future research can overcome these challenges and translate ML-driven metabolomics findings into clinical practice. As the field continues to evolve, ML-driven metabolomics holds great promise for advancing our understanding of cancer biology and improving patient outcomes.

## Data Availability

No new data were created or analyzed in this study.

## Supplementary Materials

The following supporting information can be downloaded at https://www.mdpi.com/article/10.3390/metabo15080514/s1. Table S1: Machine learning algorithms in cancer metabolomics: comprehensive summary of methods, applications, and performance across studies [5,6,8,9,10,11,12,13,58,59,60,62,63,64,65,67,68,71]. Table S2: Machine learning algorithms in cancer metabolomics, outlining their strengths, limitations, and typical applications within cancer metabolomics. Table S3: Clinically relevant ML–metabolomics findings from reviewed studies [5,6,7,10,58,59,67,70].

## Abbreviations

The following abbreviations are used in this manuscript:

MS	Mass Spectrometry;
LC-MS	Liquid Chromatography–Mass Spectrometry;
GC-MS	Gas Chromatography–Mass Spectrometry;
NMR	Nuclear Magnetic Resonance;
DDA	Data-Dependent Acquisition;
DIA	Data-Independent Acquisition;
SWATH	Sequential Windowed Acquisition of All Theoretical Fragment Ions;
ML	Machine Learning;
k-NN	k-Nearest Neighbor;
MICE	Multiple Imputation by Chained Equation;
PQN	Probabilistic Quotient Normalization;
MDFC	Maximal Density Fold Change;
RFE	Recursive Feature Elimination;
PCA	Principal Component Analysis;
t-SNE	t-Distributed Stochastic Neighbor Embedding;
UMAP	Uniform Manifold Approximation and Projection;
SNF	Similarity Network Fusion;
LASSO	Adaptive Least Absolute Shrinkage and Selection Operator;
SVM	Support Vector Machine;
NB	Naive Bayes;
RF	Random Forest;
PLS-DA	Partial Least Squares Discriminant Analysis;
CNNs	Convolutional Neural Networks;
RNNs	Recurrent Neural Networks;
AUC-ROC	Area Under the Receiver Operating Characteristic Curve;
MSE	Mean Squared Error;
LOOCV	Leave-one-out cross-validation;
TCGA	The Cancer Genome Atlas;
GEO	Gene Expression Omnibus;
SHAP	SHapley Additive exPlanations;
LIME	Local Interpretable Model-Agnostic Explanations;
SMOTE	Synthetic Minority Oversampling;
MCC	Mathews Correlation Coefficient;
TNBC	Triple-Negative Breast Cancer;
ER+	Estrogen Receptor-Positive;
BLIS	Basal-Like Immune-Suppressed;
BRCA	Breast Invasive Carcinoma;
CRC	Colorectal cancer;
PTC	Papillary Thyroid Carcinomas;
PDAC	Pancreatic Ductal Adenocarcinoma;
S1P	Sphingosine-1-Phosphate;
NAAG	N-Acetyl-Aspartyl-Glutamate;
RFS	Relapse-Free Survival;
PNT	Precancerous Normal Tissue;
SAH	S-adenosylhomocysteine;
PC	Phosphatidylcholine;
PE	Phosphatidylethanolamine;
LOWESS	Locally Weighted Scatterplot Smoothing;
OPLS-DA	Orthogonal Partial Least Squares Discriminant Analysis.
